# In vivo evaluation of a novel scaffold for artificial corneas prepared by using ultrahigh hydrostatic pressure to decellularize porcine corneas

**Published:** 2009-10-13

**Authors:** Shuji Sasaki, Seiichi Funamoto, Yoshihide Hashimoto, Tsuyoshi Kimura, Takako Honda, Shinya Hattori, Hisatoshi Kobayashi, Akio Kishida, Manabu Mochizuki

**Affiliations:** 1 Department of Ophthalmology, Tokyo Medical and Dental University, Tokyo, Japan; 2 Department of Ophthalmology, Tokyo Metropolitan Hiroo Hospital, Tokyo, Japan; 3 Institute of Biomaterials and Bioengineering, Tokyo Medical and Dental University, Tokyo, Japan; 4 Biomaterials Center, National Institute for Materials Science, Tsukuba, Japan; 5 JST-CREST, Saitama, Japan; 6 Japan Society for the Promotion of Science, Tokyo, Japan; 7 Japan Health Sciences Foundation, Tokyo, Japan

## Abstract

**Purpose:**

To evaluate the stability and biocompatibility of artificial corneal stroma that was prepared by using ultrahigh hydrostatic pressurization treatment to decellularize corneas.

**Methods:**

The porcine cornea was decellularized by two methods, a detergent method and an ultrahigh hydrostatic pressure (UHP) method. Either 1% w/v Triton® X-100 or sodium dodecyl sulfate (SDS) was used for the detergent method, and 10,000 atmospheres (atm; 7.6×10^6^ mmHg) was applied to the cornea for 10 min at 10 °C by a high-pressure machine for the UHP method. Hematoxylin-eosin staining was performed to confirm the removal of the corneal cells, and then decellularized porcine corneal stroma was implanted into rabbit corneal pockets. After eight weeks, the rabbit eyes were enucleated to examine the tissue compatibility of the implanted stroma.

**Results:**

Complete decellularization was confirmed only in corneas treated by the UHP method, and little inflammation was seen when they were implanted into the rabbit corneal pockets.

**Conclusions:**

Porcine corneal stroma completely decellularized by the UHP method has extremely high biocompatibility and is a possible corneal scaffold for an artificial cornea.

## Introduction

Injury or corneal diseases can lead to corneal opacification for which currently the only effective therapy is corneal transplantation [1]. Conditions such as corneal dystrophy, bullous keratopathy, and corneal scarring are treated by replacing the defective cornea with a clear donor cornea. Since the first human corneal transplant in 1905, corneal transplantation has been one of the most successful forms of tissue transplantation [2]. However, complications such as infection, immune rejection, and graft failure are possible, and allograft reaction has been reported to be seen in 31% of penetration keratoplasty patients. Furthermore, there is a worldwide shortage of donor corneas, due in part to many donated corneas not being able to be used because of infection.

One way to overcome these difficulties is to develop artificial corneas [3], and among the various synthetic polymers investigated for this purpose are poly(methyl methacrylate) [4], poly(2-hydroxyethyl methacrylate) [5], and poly(vinyl alcohol) [6]. Alphacor^TM^ was the first synthetic artificial cornea available commercially [7-9], but no artificial cornea has been fully successful yet. Their failure to be accepted by recipient tissue and to be invaded by the recipient’s corneal cells results in their extrusion through melting around the prosthetic rim [10] or other adverse effects such as protein adsorption, rejection with down-growth, and infection.

The engineering of cornea tissue has recently been presented as a promising solution to the limited corneal replacement with allografts. Pellegrini et al. [11] reported that the ocular surface can be reconstituted using epithelial cells cultured in vitro on a contact lens. Furthermore, Minami et al. [12] attempted to reconstitute a cornea (including the epithelium, stroma, and endothelium) in vitro by using a collagen gel culture system under an air-liquid interface. Orwin et al. [13] reported that corneal tissue could also be reconstituted in vivo by combining corneal cells and a collagen sponge. While these reports indicate the feasibility of corneal regeneration using corneal cells and collagen scaffolds, the structure and mechanical properties of their collagen gel and sponge were inadequate for an artificial cornea that can be used clinically. The mechanical properties and structure of the scaffold for corneal reconstruction must be similar to those of the natural cornea. The ideal scaffold for corneal tissue engineering would allow epithelization, endothelialization, and repopulation with autologous interstitial cells.

One strategy for preparing a scaffold is the use of decellularized tissue in which the donor cells and antigen molecules are completely removed to diminish the host immune reaction. Some groups have attempted to use porcine cornea for xenografting because it would be available in amounts sufficient to meet clinical demand [14]. Many decellularizing methods for preparing acellular tissues have been reported, and most use detergents to remove cells from tissues. Acellular tissues of the vessel, heart valve, dermis, and ligament have been successfully prepared by using Triton® X-100 [15,16], sodium dodecyl sulfate (SDS) [17,18], sodium deoxycholate [19,20], and polyethylene glycol [21] to remove the donor cells and their components. However, detergents are generally toxic and need to be washed out. Sometime, detergent treatment and the following wash-out process may lead to the denaturation of the tissue and destroy their structures.

For ideal xenografting, the cellular immune reaction should be decreased by removing donor cells from the cornea, but the corneal superstructure should be maintained to keep the cornea transparent. In general, the transparency of the cornea is explained by a lattice theory of the corneal materials in which the corneal superstructure is an optically clear lattice of regularly aligned collagen fibrils. Thus, the ideal decellularization process would be one that removes all the cell components without destroying the corneal superstructure.

**Figure 1:**
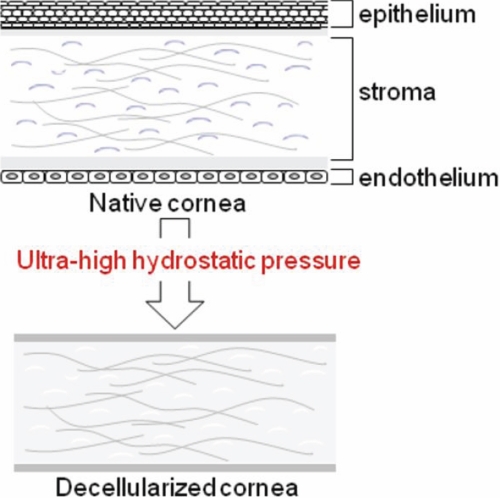
Concept of decellularization using ultrahigh hydrostatic pressure. Ultrahigh hydrostatic pressure (UHP) treatment removes all the cells of the native cornea (epithelial cells, keratocytes, and endothelial cells).

Several methods have been reported to be effective for decellularizing corneas [22-26], and decellularizing corneal tissues have been shown to be biocompatible. However, their mechanical characteristics still need to be improved. We recently developed a novel physical process that uses ultrahigh hydrostatic pressure (UHP) technology to decellularize tissue without using detergents [27] (Figure 1), and in the work reported here, we compared its use with that of a detergent method in the decellularization of porcine cornea. Using decellularized porcine cornea, an implantation experiment into the rabbit eye was performed to see if we could apply the decellularized porcine cornea for xenografting as an artificial cornea.

## Methods

### Materials

Porcine eyes were purchased from Shibaura Zoki Co., Ltd. (Tokyo, Japan). Japanese white rabbits were purchased from Kitayama Labes Co., Ltd. (Nagano, Japan). Dextran (molecular weight=70,000 g/mol) was purchased from Tokyo Kasei Kogyo Co., Ltd. (Tokyo, Japan). SDS was purchased from Wako Co., Ltd. (Osaka, Japan). Triton® X-100 was purchased from Sigma-Aldrich Co., Ltd (Tokyo, Japan). Phosphate buffer saline (PBS) was purchased from Invitrogen Co., Ltd. (Tokyo, Japan). Endothelial growth medium (EGM-2) was purchased from Sanko Junyaku Co., Ltd. (Tokyo, Japan).

### Preparation of porcine cornea

The entire cornea was removed from the eye, washed with PBS containing penicillin (100 units/ml) and streptomycin (0.1 mg/ml), and stored at 4 °C in PBS containing these antibiotics and dextran (3.5% w/v) until the experiments were performed.

### Chemical decellularization

Corneas were immersed in a 1% w/v solution of either Triton® X-100 or SDS at 37 °C for 24 h, washed with PBS containing penicillin (100 units/ml) and streptomycin (0.1 mg/ml) for another 24 h, and then subjected to hematoxylin-eosin (H-E) staining for histological study.

### UHP decellularization

Corneas were pressurized at 10,000 atm for 10 min at 10 °C by using a high-pressure machine (Kobe Steel, Ltd., Kobe, Japan), washed under air containing 5% CO_2_ by continuous shaking for 72 h at 37 °C in an EGM-2 medium containing DNase I (0.2 mg/ml), antibiotics, and 3.5% w/v dextran, and then subjected to hematoxylin-eosin (H-E) staining for histological study before they were used for transplantation.

### Histological study

Native and decellularized corneas (five of each) were fixed for 24 h in a 10% neutral buffered formalin solution at room temperature. They were then cut, stained with H-E, and observed with an optical microscope.

### Measurement of residual DNA content

After 20 mg of each freeze-dried decellularized cornea was digested at 55 °C for 12 h in 0.5 ml of a tissue lysis buffer containing 50 mM Tris-HCl, 50 µg/ml proteinase K, 1% w/v SDS, 100 mM NaCl, and 20 mM disodium EDTA, the DNA content was calculated from the difference in the absorbance at 260 nm measured before and after extracting DNA with phenol and chloroform and precipitating it with ethanol. Five corneas were used for each group.

### Measurement of residual GAG content

Glycosaminoglycans (GAGs) or mucopolysaccharides such as hyaluronic acid and chondroitin sulfate help maintain the structure of connective tissues, so a decreased GAG content would indicate destruction of tissue structure. We therefore used an Alcian blue assay to measure the residual GAG in the tissue. After 20 mg of each freeze-dried decellularized cornea was digested at 65 °C for 24 h in a papain solution (100 mM sodium acetate buffer, 0.5 mg/ml papain, 0.5 mM disodium EDTA), Alcian blue was added and a microplate reader was used to measure the absorbance at 600 nm. The GAG was calculated from the absorbance by using chondroitin sulfate standard solutions. Five corneas were used for each group.

### Statistical analysis

Measurements of residual DNA and GAG content were performed three times. Mean±SD values were calculated. Data were analyzed statistically by Student’s *t*-test. A p<0.05 was regarded as significant.

### Preparation of decellularized porcine cornea

After physiologic saline was injected into the vitreous humor of a porcine eyeball to raise the intraocular pressure, a microkeratome was used to prepare a corneal flap 160 µm thick. The corneal flap was treated with ultrahigh hydrostatic pressure three days before the transplantation, and it was stored in an EGM-2 medium at 4 °C until transplantation. Corneal discs 2 mm in diameter were prepared with a corneal punch.

### Transplantation of decellularized porcine cornea into rabbit corneal stroma

**Figure 2:**
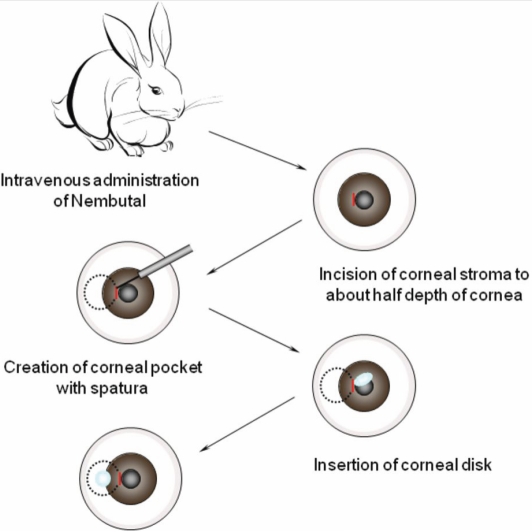
Procedure of transplantation of decellularized porcine corneal disc into a rabbit corneal pocket. The recipient rabbit is anesthetized with intravenous Nembutal and topical Xylocaine, a corneal pocket is made with a spatula, and a decellularized or native porcine corneal disc is inserted into the corneal pocket.

Decellularized porcine cornea was transplanted into the left eye of Japanese white rabbits (female, 3 kg and 12 weeks old) according to the ARVO Statement for the Use of Animals in Ophthalmology and Vision Research. All animal experiments were approved by the ethical committees for animal welfare of Tokyo Medical and Dental University (Tokyo, Japan) and National Institutes for Materials Science (Tsukuba, Japan). The corneas of the recipient animals (n=11) anesthetized with intravenous Nembutal^TM^ (Dainippon Sumitomo Pharma Co., Ltd., Osaka, Japan) and topical Xylocaine^TM^ (AstraZeneca, Osaka, Japan) were incised with a surgical knife to about half the depth of the corneal stroma, tangent to the pupil at four positions 90° apart around the edge of the pupil (3, 6, 9, and 12 o’clock). Stromal pockets were then formed by inserting a spatula into each incision, directing the inserted edge of the spatula toward the corneal limbus, and using it to separate the stromal layers. A decellularized porcine corneal disc was put into three of the pockets, and a non-decellularized one was put into the other pocket as a positive control (Figure 2). Eight weeks after the transplantation, the left eye was enucleated and the cornea was stained with hematoxylin and eosin for histological study.

## Results

### Decellularization

**Figure 3:**
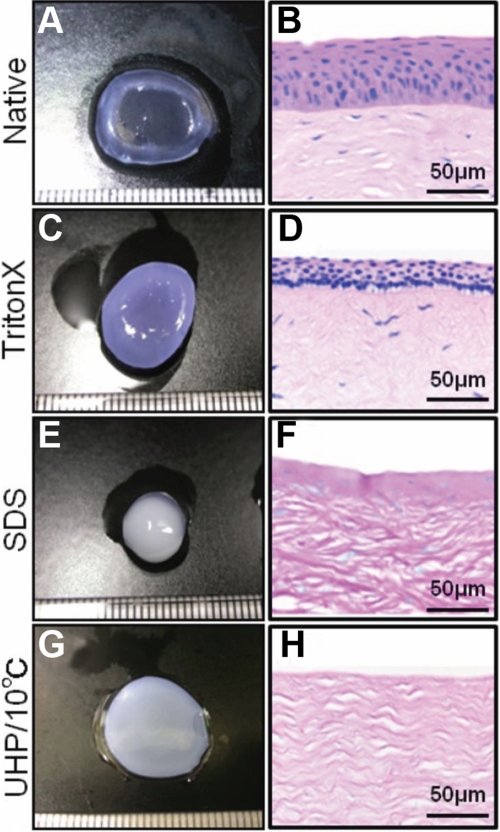
Representative photographs and H-E stained sections of the porcine corneas decellularized by various methods. The left column shows photograph of native cornea (**A**), cornea treated with Triton® X-100 (**C**), cornea treated with SDS (**E**) and cornea decellularized by UHP (**G**). The right column shows H&E stained section of native cornea (**B**), cornea treated with Triton® X-100 (**D**), cornea treated with SDS (**F**) and cornea decellularized by UHP (**H**). Epithelial cells and keratocytes are seen in the corneas treated with Triton® X-100 or SDS but not in the cornea treated with UHP. Scale bar, 50 µm.

After a porcine cornea was immersed in a 1% w/v solution of Triton® X-100 for 24 h and then washed with PBS for 24 h, it was cloudy and more than five times thicker (Figure 3C) than it was before treatment (Figure 3A). Comparing an H-E stained section of the native cornea (Figure 3B) with that of a cornea treated with Triton® X-100 (Figure 3D), one sees loosening of the collagen fibrils in the corneal stroma and shrinkage of the epithelial layer in the treated cornea and also that few cells of the cornea were removed by the treatment. The cornea treated with SDS (Figure 3E) was not swollen as much as the one treated with Triton® X-100 (Figure 3C), but it was smaller, its surface was melted, and its interior was extremely cloudy because the nuclear materials of the disrupted cells remained (Figure 3F). Thinning of the epithelial layer and disordering of the superstructure of collagen fibrils in the stroma were also observed in the corneas treated with the detergents.

Although the porcine cornea treated with UHP was also extremely cloudy and slightly swollen after washing in an EGM-2 medium (Figure 3G), H-E staining showed the absence of cells in the epithelium and stroma and the maintenance of the superstructure of collagen fibrils in the stroma (Figure 3H). These results indicate that the UHP method is useful for decellularizing the porcine cornea without destroying its structure.

### Confirmation of decellularization

The DNA content of the corneal tissues treated with Triton® X-100 and SDS were 2.32±0.28 and 1.16±0.21 µg/mg, respectively. They were significantly lower than the DNA concentration of the native cornea (3.46±0.18 µg/mg) but much greater than zero. On the other hand, the DNA concentration of the porcine corneal tissues treated with UHP is almost zero (0.12±0.02 µg/mg), which indicates complete removal of cell components from the corneal tissue. (Table 1)

**Table 1:** Confirmation of decellularization.

**Sample**	**DNA content mean±SD (µg/mg dry wt)**	**GAG content mean±SD (µg/mg dry wt)**
Native cornea	3.46±0.18	223.0±19.1
Triton® X-100	2.32±0.28*	169.9±8.33*
SDS	1.16±0.21*	38.6±3.01*
UHP/10 °C	0.12±0.02*	201.3±10.1

The DNA and GAG content of the corneas decellularized by various methods were measured. These results indicate that only the corneas treated with UHP lost cells without losing GAG-collagen interactions. Data are expressed as the mean±SD; n=5 in each group. The asterisk indicates a p<0.05.

### GAG content

The GAG content of the corneal tissues treated with Triton® X-100 and SDS were 169.9±8.33 and 38.6±3.01 µg/mg, respectively, significantly lower than the GAG content of native cornea (223.0±19.1 µg/mg), indicating that the structure of the connective tissues is not maintained in detergent-treated corneas. The GAG content of the corneal tissues treated with UHP, on the other hand, is almost the same as that of native cornea, which means the connective tissue structure was not destroyed by the UHP treatment. (Table 1)

### Transplantation of decellularized porcine cornea into rabbit corneal stroma

**Figure 4:**
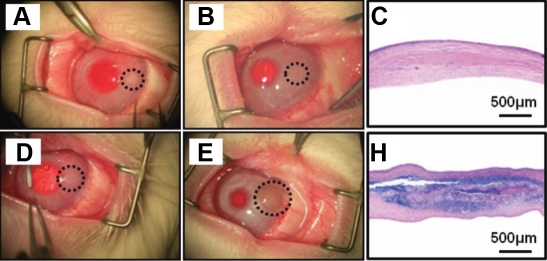
Representative photographs and H-E stained sections of transplanted porcine corneal discs. The left column shows photograph of decellularized (**A**) and native (**D**) porcine corneal disc just after transplantation into rabbit corneal pockets. The middle column shows photograph of decellularized (**B**) and native (**E**) porcine corneal disc eight weeks after transplantation. The right column shows H&E stained section of decellularized (**C**) and native (**F**) porcine corneal disc eight weeks after transplantation. Decellularized corneal discs caused slight inflammation and recovered transparency whereas native corneal discs caused severe immune reactions and remained cloudy. Scale bar, 500 µm.

To evaluate the possibility of using porcine corneas decellularized by the UHP method as a substitute for corneal stroma, we implanted them in corneal stromal pockets in rabbits and implanted native porcine corneas as positive controls. The native implanted corneal discs were fairly clear just after the operation (Figure 4D), but one week later, blood vessels were seen in them and they began to become cloudy (data not shown). The native donor tissue was extremely cloudy four weeks after implantation, and eight weeks after implantation, many vessels were observed around it, indicating the occurrence of an immune reaction (Figure 4E). The histological section showed the infiltration of neutrophils and macrophages in and around the donor tissue and also showed the formation of a cell layer (Figure 4F). On the other hand, in the decellularized cornea group, the donor tissue appeared very cloudy just after implantation (Figure 4A) but began to become transparent one week later (data not shown). Two weeks later, the donor tissue in the decellularized cornea group was completely transparent and could not be recognized without a microscope. The transparency was kept until eight weeks after the implantation, and no vessels were observed (Figure 4B). The histological section showed minimal inflammation around the donor tissue, which indicated that no immune rejection occurred (Figure 4C).

## Discussion

In this study, we compared two methods of tissue decellularization and demonstrated the extremely high biocompatibility of porcine corneal discs decellularized by the UHP method.

Corneal decellularization was performed by chemical and physical methods. Triton® X-100 and SDS, which have often been used in decellularization protocols [28], were used for chemical decellularization. These detergents caused the corneas to swell and become irreversibly cloudy. They did not become transparent again even when they were treated with glycerol (data not shown). The obvious decrease of GAG content also indicates the disruption of the corneal superstructure. These results suggest that the superstructure of the cornea was strongly denatured by Triton® X-100 and SDS. The UHP method, on the other hand, removed all corneal cells. Although corneas treated with UHP were extremely cloudy just after they were treated, they became transparent again when treated with glycerol (data not shown). The glycerol treatment dehydrated the UHP-treated cornea, playing the role normally played by the Na^+^-K^+^ pump of the endothelial cells. The results of the histological study and the measurement of GAG content also indicate the maintenance of the corneal superstructure. The swelling seems to be caused not by the disruption of corneal superstructure but by the lack of the pumping function of the endothelial cells.

In the transplantation study, only native corneas and corneas decellularized by the UHP method were used. Triton® X-100 and SDS did not remove all the cells from the corneas. It was obvious that corneas with residual cells would cause a severe immune reaction to the recipients like native corneas. Moreover, SDS decreased the mechanical strength of the corneas so much that they could not be inserted into stromal pockets. Therefore, we used native corneas and corneas decellularized by the UHP method from the viewpoint of animal protection.

The transplantation of decellularized porcine corneas into rabbit corneal pockets induced little immune reaction whereas native corneas caused severe inflammation. This decellularization method involves two processes, disruption of the cells, bacteria, and viruses by ultrahigh pressurization [29,30] and removal of the residues of cellular components by washing in a culture medium. We previously reported the successful decellularization of porcine heart valve and trachea by this method [27]. In the transplantation experiment, the decellularization process removed all the corneal cell components including bacteria and viruses. The native cornea tissue may have been rejected not only of xenografting but also because of infectious pathogens. Although many countries are running short of donated corneas, some are rejected because of infection. The UHP method may reproduce unusable corneas from infected corneas by removing the pathogens.

In conclusion, we have developed a corneal decellularization method using UHP technology. The decellularized corneal tissue did not cause immunological rejection in a xenograft transplantation model. These results indicate that decellularized cornea stroma that was prepared using UHP technology is a possible scaffold for an artificial cornea.
